# Society Proceedings

**Published:** 1898-08

**Authors:** 


### SOCIETY PROCEEDINGS.

### CHICAGO VETERINARY SOCIETY.

THE meeting was called to order at 8 P.M., June 9, 1898, by Dr. Walker, President. Dr. Walker stated that he had been unable to see the directors of the Chicago St. Andrew's Society in regard to the rental of the club-room we are now occupying. He promised to see them before the October meeting.

The report of the Treasurer showed $14.03 in the treasury. No report from the Secretary.

Dr. Griiner read a paper relative to the subject of "Examination of Horses for Soundness." Much interesing discussion took place from the doctor's production and opinions. On motion, the discussion was closed.

The following members have severed their connection with the Society: Drs. E. Jentzsch, James Flemming, J. A. Bovett, Jr., Harry J. Stewart, D. W. McKillip, A. R. Wake, J. McBirney, R. H. Tracy, L. Clark, W. J. Stewart, C. H. Zink.

On motion, adjournment.

L. CAMPBELL, D.V.S.,
Secretary.

### *Dr. Griiner's Paper.*

My subject for this evening is an extensive one, and I am afraid that I am not able to give each the attention it should have; but if I can only arouse a lively discussion on the different diseases, among my fellow-members, I shall be very grateful. The essay is confined to the foot, and includes low ringbone, side-bones, coronitis, calks, quarter-cracks, toe-cracks, navicular arthritis, keratoma, contraction, laminitis, corns, dropped sole, thrush, seedy toe, canker, and quittor.

*Low ringbone* is a disease affecting the distal interpharyngeal articulation (or coffin-joint). It appears as an exostosis, generally on the anterior of the limb at coronet, but may appear somewhat laterally, and generally involves the whole joint; whether causing lameness or not, I would consider the animal unsound. We may find some enlargement on the os corona not involving the joint, also called false ringbone; this I would not consider an unsoundness.

*Side bones*, or ossification of the lateral cartilage, when they become hard and unyielding, are found on the posterior lateral aspect of the foot. There is generally very little lameness present. Would consider it an unsoundness.

*Coronitis*, or *villitis*, inflammation of the coronary band, not very often met with; recognized by heat, pain, and fulness of coronary band; is an unsoundness.

*Calks*, or treads on the coronet, received from the animal stepping on himself with shoe on opposite foot; the injury may be slight or very grave; unsoundness would therefore depend upon injury to the part.

*Quarter-cracks* are splits or fissures on the hoof occurring at the quarter, mostly found in front feet. They may be superficial or deep. When deep there is a complete fracture of the crust extending into the sensitive lamina, in which case I would consider it an unsoundness; when superficial, however, horse may be considered sound.

*Toe-cracks* may be considered under the same head as quarter-cracks, the difference being the cracks occurring at toe. If crack extend through the full length of the hoof I would call it an unsoundness.

*Navicular arthritis* is a disease of the navicular joint. Opinions differ somewhat in regard to the point of origin; some place it in the perforans tendons, others on the gliding surface of the os navicular, and still others say positively it originates in the synovial bursæ; but that matters but little, and if the examining veterinarian's diagnosis is correct, he need not hesitate in pronouncing the animal unsound.

*Keratoma*, also termed keratopholocele, develops generally on the inner and anterior extremity of the wall of the toe at the white line, and may extend as high as the coronet, leaving a marked depression on the pedal bone. This is an unsoundness.

*Contraction* is a name applied when hoofs become narrow at heels, and is often associated with thrush and navicular arthritis. This is, in my opinion, an unsoundness.

*Seedy toe* may be termed localized laminitis. The wall becomes detached from the sensitive lamina, leaving a cavity, where we find a mealy deposit. It may appear at toe or quarter. For slow work such a horse may be all right, but I would be inclined to pronounce him unsound.

*Corns* are located at the bars or heels of the foot. If a bruise, it is termed "dry corn;" if suppurating, a "suppurative corn," involving the sensitive lamina, mostly seen in front feet. A suppurative corn is an unsoundness.

*Thrush* is a disease confined to the commissure of the frog, mostly seen in hind feet. If seen in front feet, look for some other disease. If front feet are affected, I consider it an unsoundness; if hind feet, not.

*Laminitis*, also known as founder, is inflammation of the sensitive lamina; whether the disease is acute or chronic, animal should be pronounced unsound.

*Dropped sole* generally occurs from the effect of laminitis; is a condition where the sole descends, and part of the os pedis rotates downward. Such an animal may perform slow work, but is unsound.

*Canker* is a morbid growth affecting the sensitive sole, frog, and walls; it is a chronic proliferation of the papillæ of the rete, producing a bad-smelling, greasy, yellowish-gray fluid, and is an unsoundness.

*Quittors* are fistulous tracts usually opening at coronet. We also have cartilaginous quittors affecting the lateral cartilage; sometimes the subcoronary tissue only is involved, but there may be several fistulous tracts, all involving sensitive laminæ. This is, in my opinion, an unsoundness.

*Discussion.*

Dr. Quitman: In speaking about corns, the essayist remarked that a suppurating corn was decidedly unsound, but avoided corns in general. Does he consider a horse that seems to have corns, but does not go lame, as unsound?

Dr. Griiner: I mentioned dry corns. I did not state whether they are sound or not. If removable, the horse may be called sound. I would not consider nor pass any as sound, as it is hard to keep shoes on horses with corns.

Dr. Baker: I would like to know as to whether, in the absence of any soreness, a veterinarian is justified, with the view of simply looking for them, to pare away the hoof in the seat of a corn, assuming that there were no indications creating suspicion that there were any. I know some practitioners do make it a practice to pare away the hoof, with this object, in every case. If they find a discoloration they condemn the horse.

Dr. Quitman: I would say, as far as I am concerned, in examining a horse for soundness, I invariably examine the horse for corns, with a knife, paring the inner, and, in city horses, also the outer bar and sole. In my opinion, however, if a man is going to condemn a horse for corns he will have to condemn about three-fourths of the horses. Even country horses in the city will develop corns, caused by the hardness of the pavement or from the difference in the stabling. It depends upon the severity of the corn whether they should be condemned. I invariably, however, examine every foot for self-protection, if for nothing else.

Dr. Griiner: I would like to ask Dr. Quitman whether he takes the shoes off in making his examination; that is, if he has to examine a horse with shoes on?

Dr. Quitman: It depends upon the web and style of the shoe; also on general conditions. I often have the front shoes removed. If it does nothing else it impresses the client that I make a very careful examination. I make it a rule to point out every little blemish to the owner for my own safety. If the corn is of a deep purple color, and extends deeply between the bar and the wall, I reject the horse. If, however, the horse does not show any lameness or tenderness, I would, as a rule, pass him, although he has a corn. It depends mainly upon the discoloration, etc.

Dr. Griiner: Horses shod with rolling motion or rubber pads are, as a rule, unsound, and I would recommend particular care in examining such horses.

Dr. Quitman: Does the essayist consider a horse unsound having a Whitman and Barnes rubber shoe on?

Dr. Griiner: Not necessarily. I would examine him carefully, having his shoes taken off.

Dr. Robertson: I must confess that I have not been as particular heretofore in examining the feet for corns. I rely as a rule, on the external conformation of the wall and appearance of the foot in general. In my opinion, we are not supposed to pare into the sole of the foot, except we have just cause of suspicion, the same as we are not supposed to go into a bowel that may be weakened from some digestive trouble. External appearances will caution an experienced veterinarian for closer examination. Then he can use his own judgment as to the horse being sound or unsound.

Dr. Campbell: Would you reject a horse with a slight corn?

Dr. Robertson: That depends upon the conformation of the foot.

Dr. Hughes: Regarding the subject of corns, something like the subject of ringbone and many other conditions touched upon, we have to use a very considerable lot of judgment in passing it for soundness or unsoundness. In my opinion corns are not produced by the shoe, but by the method of the shoeing. We hear of horses from the country that have never been shod having corns. However, this is an unreasonable statement. When a man runs across such a corn, it is merely an accidental bruise, or staining of a healthy quarter, and should be considered in my opinion as sound. Any corn that looks like a long-standing one should be pronounced as unsound. I would like to have the question of ringbone discussed to-night. In my opinion, the subject of ringbone in examination of horses for soundness will cause more annoyance than anything else. For instance, a heavy draught-horse five years old that is perfectly sound as far as his movements are concerned. When we come to examine his os corona we find on the posterior lateral aspect a marked angularity. The anterior as well as the internal lateral aspects are smooth. Is that horse sound? I hold that a man who finds a marked angularity at that point is perfectly justified in condemning the animal, as he will have a ringbone in the future. Anyone can determine a pronounced ringbone, but I would like to hear the opinion of the members, as undoubtedly they have come across such irregularities.

Dr. Baker: I think it is unjust that a horse with rough pasterns should be condemned. My experience has taught me that they are no more liable to develop ringbones than others, and therefore I think it is unjust to ourselves and to commerce to condemn a horse with rough pasterns, provided there is no thickening or other cause for ringbone. In regard to examination for corns, where there is no suspicion I do not pare away the hoof, nor do I condemn a horse with slight discoloration without any lameness.

Dr. Hughes: Regarding Dr. Baker's statement of angularity, I never condemn a horse that is all angular; but when I find a horse that has pronounced roundness of the joints and angular pasterns, I say there is something wrong, and reject the horse.

Dr. Merrillat: My experience regarding rough-jointed horses and rough pasterns is, that you will usually find a horse that has rough pasterns is not rough-jointed, and I fully believe that such a condition should be looked at with suspicion. I have examined more than eight hundred cavalry horses this last week, and condemned as many as forty with these enlargements, as I considered them as absolutely unsound.

Dr. Robertson: What is your opinion as to soundness of a horse with one foot smaller than the other?

Dr. Hughes: I think such a horse should be in every case rejected, as he will sooner or later, especially in the city, go lame, and invariably on the smaller foot.

### GENESEE VALLEY VETERINARY MEDICAL ASSOCIATION.

THE regular semi-annual meeting was held at the Livingston Hotel, Rochester, N. Y., on July 21, 1898. The meeting was called to order at 11 A.M., by the President, Dr. A. Drinkwater, of Rochester. At the roll-call the following members responded: Drs. A. George Tegg, L. R. Webber, E. Knight, and J. C. Mackenzie, of Rochester; O. B. French, Honeoye Falls; J. H. Taylor, Henrietta; N. N. Lefler, Geneseo; W. B. Switzer, Williamson; P. J. Johnson, Sodus; G. C. Kesler, Holly; Thomas Flood Gorham; and E. H. Nodyne, Clyde.

The minutes of the last meeting and the Treasurer's report were read and approved. Dr. William Hunter, of Dansville, was proposed and elected a member.

The Committee of Incorporation reported that the Association had been incorporated in accordance with the laws of the State governing incorporation of societies. They also exhibited a specimen of a membership certificate, which was accepted. Two very interesting papers were read by Drs. Switzer and Lefler, and several specimens were exhibited and cases reported, all of which led to a long and interesting discussion.

The following members are to prepare papers for the next meeting: Drs. Nodyne, Knight, Kesler, Drinkwater, and Mackenzie. The meeting then adjourned.

A. George Tegg,
Secretary.

### MINNESOTA TO THE FRONT—A SUGGESTION.

The veterinarians of Minnesota have recently adjourned one of the most successful State Association meetings that it has been the privilege of the writer to attend. For many years it seemed impossible to maintain a State association in Minnesota. Several were formed and soon failed. Perhaps the explanation of the success of our recent meeting may offer some points of interest and possibly suggestions for other associations that are struggling with the old problem of how to get veterinarians to join and attend.

Most of the veterinarians who attended our recent meeting at Faribault reached that city by 11 A.M., July 14th. We assembled at once, and routine business matters were all disposed of by noon.

The entire afternoon was given to clinical work. Dr. Hay, Secretary, who lives at Faribault, had prepared a splendid clinic, which kept us busy Thursday afternoon and Friday forenoon. The operations were distributed fairly and evenly among the members. The following operations were demonstrated at these two clinics:

Removal of molar and operation on inferior maxilla; three tendons fired by different operations; two cunean tenotomies by different operators; one urethrotomy with removal of calculus; four lower neurectomies; removal of shoulder fibroid; diagnosis of obscure lameness by means of cocaine; two caudal myotomies for straightening by different operators; removal of two nasal tumors; puncture cautery on two spavins by different operators.

We spent Thursday afternoon with the surgical work, and were all busy. During the evening we attended to the literary part of the programme, which was somewhat unique. There was nothing presented on this programme that could be called a formal paper, excepting possibly a report of the Committee on Infectious Diseases. The entire evening until eleven o'clock was spent in what might be called an experience meeting. Every member came prepared to report one or more experiences that might be of value to fellow practitioners. Unusual cases, mistakes in practice, errors in diagnosis, etc., were reported in abundance. Questions were asked, information given, and everyone went to the hotel shortly before midnight full of enthusiasm and thoroughly satisfied. After this formal adjournment quite a number of the members went to the office of Dr. Hay, Secretary, and spent several hours more, holding an informal session, discussing cases and experiences.

The following forenoon was devoted again to surgical clinic. As a rule, but one operation was on the programme at a time, so that everyone could get the benefit from personal observation and by questioning the operators.

All this surgical work was uniformly well done. This was especially noticeable in view of the fact that so large a number of operators took part.

The success of this meeting may give useful suggestions to other secretaries who would score success such as Dr. Hay has made for us.

I would not suggest this programme as one to be regularly followed, but I would draw attention to two points, viz., a large portion of the programme should always be practical, and there should be variety and something original and unique once in a while.

There are a great many intelligent veterinarians in each State who have a lively interest in corns and spavins, and do not care whether the hog-cholera bacillus is twin brother to the swine plague bacillus, or only a very distant relative, nor which one, if either, is motile. I would not underestimate the value of purely scientific papers, but let there be a limit and make programmes distinctly practical.

M. H. REYNOLDS, D.V.M.
St. Anthony Park, Minn.

### MINNESOTA STATE VETERINARY MEDICAL ASSOCIATION.

THE second semi-annual meeting of this Association was held at Faribault, Minn., on July 14 and 15, 1898. In the absence of the President, Vice-President Reynolds called the meeting to order at 11 A.M. at the Council chamber. Those in attendance were: Drs. W. Amos, Owatonna; J. J. Armaud, Minneapolis; S. D. Brimhall, Minneapolis; C. C. Lyford, Minneapolis; B. A. Pomeroy, St. Paul; R. Price, St. Paul; J. N. Gould, Worthington; J. A. Hanish, Lake City; J. W. Gould, Fairmont; K. J McKenzie, Northfield; M. H. Reynolds, St. Anthony Park; S. H. Ward. St. Cloud; H. C. Lyon, Hutchinson; James Nicholson, Pipestone; A. F. Lees, Red Wing; L. Hay, Faribault; George Milligan, Faribault.

The reports of the Secretary and Treasurer were read and adopted, and other routine business transacted, after which the members adjourned for dinner.

In the afternoon the members met at L. Miller's livery stable, where Secretary Hay previously gathered in a number of interesting surgical cases, which were operated on as follows: Removal of diseased molar and operation for dental fistula of the lower jaw, Dr. J. W. Gould, Worthington. Caudal myotomy for straightening crooked tail, Dr. R. Price, St. Paul. Caudal myotomy, Dr. K. J. McKenzie, Northfield. Cunean tenotomy, Dr. S. D. Brimhall, Minneapolis. Cauterization of tendons, Dr. L. Hay, Faribault. Cauterization of tendons, Dr. B. A. Pomeroy, St. Paul. Operation on horse's shoulder for the removal of a fibrous growth, Dr. M. H. Reynolds, St. Anthony Park. The meeting then adjourned until after supper, when it was called to order again about 8 P.M. Drs. Butler and Keyes, of Minneapolis, and H. C. Lyons, of Hutchinson, and James Nicholson, of Pipestone, were elected members of the Association, after which a number of interesting cases were reported by the different members.

Friday morning the members assembled again to witness the following clinical programme: Urethrotomy for removal of a cystic calculus, Dr. M. H. Reynolds, St. Anthony Park. Cunean tenotomy, Dr. R. Price, St. Paul. Cauterization of tendons, Dr. L. Hay, Faribault. Removal of sebaceous cysts, Dr. C. C. Lyford, Minneapolis. An adjournment for dinner was then taken.

Clinical work was resumed at 2 P.M. Excision of fibroid tumor, Dr. C. C. Lyford, Minneapolis. Lower plantar neurectomy, Dr. M. H. Reynolds, St. Anthony Park. This finished the clinical programme of the meeting.

A vote of thanks was tendered Dr. Hay, of this city, for the abundant clinical material secured and for the effort in making this the most successful meeting ever held in the State. A vote of thanks was also tendered to Mr. L. F. Miller, of this city, for his courtesy in supplying suitable quarters for the holding of the clinics, and also to the proprietor of the Commercial Hotel for his hospitality and courtesy to the members of the Association. The meeting then adjourned.

L. HAY, V.S.,
Secretary-Treasurer.

### NEW YORK STATE VETERINARY MEDICAL SOCIETY.

TITLE of papers to be read at the annual meeting, September 14 and 15, 1898, at the Hotel Metropole, Forty-second Street and Broadway, New York City: "Glanders and its Relation to Mortality," Dr. James Law, New York State Veterinary College; "Nail Wounds of the Feet in Horses," Dr Roscoe R. Bell, American Vetrinary College; "Notes on Tooth Tumors," Dr. W. L. Williams, New York State Veterinary College; "Osteoporosis," Dr. George Berns, Brooklyn, N. Y.; "The Science *versus* the Art of Veterinary Surgery," Dr. Robert W. Ellis, New York City; "Hydrotherapy in Domestic Animals," Dr. W. L. Williams, New York State Veterinary College; "A New Treatment of Milk Fever," Dr. Olaf Schwartzkopf, Flushing, L. I., New York; "Streptococcus Injection in Domestic Animals," Dr. V. A. Moore, New York State Veterinary College; "Some Experiments with Antiseptics," Dr. P. A. Fish, New York State Veterinary College; "Notes on the Embryology of Domestic Animals," Dr. Simon H. Gage, New York State Veterinary College; "Relation of the Ligamentum Nuchæ to the First Cervical Vertebra in the Horse," Dr. G. S. Hopkins, New York State Veterinary College; "A Simple Test for the Detection of Albumin in Urine," Dr. P. A. Fish, New York State Veterinary College.

### MISSOURI VALLEY SOCIETY PROCEEDINGS.

THE Iowa State Veterinary Medical Association will hold a joint meeting with the Nebraska State Association on Monday, September 5, 1898, the day before the convening of the U. S. V. M. A., and possibly the Missouri Valley may meet with them. The annual meeting of the Iowa Association will be held in Des Moines in the fall or winter. Officers of this Association are as follows: President, S. H. Johnson, Carroll; Vice-Presidents, G. W. Scott, Independence, and D. G. Parslow, Marshalltown; Secretary-Treasurer, John E. Brown, Oskaloosa. Delegates to U. S. V. M. A. meeting, Drs. P. O. Koto, Forest City; H. E. Talbot, Des Moines. Board of Censors, Drs. W. H. Austin, Newton; D. H. Miller, Harlem; J. H. McLeod, Charles City.

### MISSOURI VALLEY VETERINARY MEDICAL ASSOCIATION.

*Officers*—President, S. E. Bennett; First Vice-President, R. C. Moore; Second Vice-President, J. C. Milnes, Kansas City, Mo.; Secretary and Treasurer, W. A. Heck, South St. Jospeh, Mo.

The Missouri Valley Veterinary Association fixes its place of annual meeting at Kansas City by insertion in their by-laws. Its membership territory covers a radius of seventy-five to one hundred miles. At present its membership reaches St. Joseph, Mo., on the north, Galena, Kansas, on the south, from Eldorado, Emporia and Concordia, Kansas, on the west, to Chillicothe and Clinton, Mo., on the east, with an occasional member along the valley farther away.

From time to time the drift of thought has given consideration among a number of the more ambitious veterinarians of the West toward the creation of a Western veterinary association composed of the Dakotas, Minnesota, Nebraska, Iowa, Kansas, Missouri, Oklahoma, and other States. The proposition will be further considered at Omaha.

### ASSOCIATION OF VETERINARY FACULTIES OF NORTH AMERICA.

SECRETARY GILL has sent out the programme for this meeting.

A special committee will report on the advisability of forming a National Examining Board, co-operation with State associations, the securing of more uniform laws, the standard of requirements to be established by the boards rather than by fixed laws, the interchange and recognition of certificates where satisfactory requirements exist.

Dr. D. E. Salmon of the Columbian University Veterinary Department, will consider the subject of "Uniform Standard of Entrance Examination."

The value and need of an Interstate Examining Board whose certificate would be accepted by the boards of all States will be considered by Drs. W. Horace Hoskins, for Pennsylvania; James Law, for New York State; A. W. Clement, for Maryland; H. J. Detmers, of Ohio; F. H. Farmer, of North Dakota; Charles McCulloch, of Virginia, and M. H. Reynolds, of Minnesota.

A proposition will be acted upon of admitting members of State Examining Boards to the Association of Faculties.

President Pearson expects an unusual interest and attendance and the thorough consideration of many problems connected with higher veterinary education.

### PENNSYLVANIA STATE VETERINARY MEDICAL ASSOCIATION.

THE Pennsylvania State Veterinary Medical Association will hold its semi-annual meeting at Pittsburg on September 20, 1898.

Secretary Rhoads reports an interesting programme and indications of a large attendance.

Dr. J. C. McNeil, of Pittsburg, with a number of other veterinarians of Allegheny County, will look after the welfare of those in attendance.

President Jobson expects a large attendance of Western members, and the better practice among veterinarians will be largely conducive to this end.

Many veterinarians in the western part of the State not identified in membership should be applicants at this meeting.

Those from the eastern part of the State will find an available ticket for this meeting in the thousand-mile book of the Pennsylvania Railroad, issued at two cents per mile, good for one year over any of the branch roads.

A party ticket for ten may be obtained from Philadelphia at two cents per mile, making the cost going and coming $14.16. To secure this favored rate all must go and come at the same time.

The 8.30 A.M. train by the Pennsylvania Railroad reaches Pittsburg in time for supper. The 8.50 P.M. train arrives in time for breakfast the following morning. The 12.25 P.M. train reaches Pittsburg at 11 P.M. The 10.20 P.M. train arrives at 8 A.M. the following morning. Those desiring the trip by day should take the 8.30 A.M or 12.2 5P.M. The evening trains have sleeping cars attached, and the train leaving 10.27 P.M. is open for the reception of passengers an hour earlier.

Many matters of the greatest importance will be taken up, and all are assured of a good time.

U. S. V. M. A.

Many fresh specimens from the slaughter-houses of Kansas City will be shown in connection with the consideration of meat-inspection.

The joint meeting of the Nebraska and Iowa State organizations at Omaha will be purely social in character.

The members of the Nebraska Association will furnish the clinical material for the meeting at Omaha.

Dr. James Law will contribute to the discussion of the subject of "Meat-Inspection.'"

Drs. Joseph Hughes, of Chicago, and John W. Connoway, of Columbia, Mo., will contribute papers to the meeting not down on the programme.

Mr. Charles R. Squibb, of Brooklyn, will offer a paper of much moment and importance for the consideration of those present.

A very large Western attendance is indicated. Better times, brighter hopes and prospects are aiding the meeting.

The clinical features of the meeting, with practical demonstrations, should prove an interesting and instructive portion of the convention's work:

"Caudal Surgery," by Dean Gill, of the New York College of Veterinary Surgeons.

"Ovarectomy in the Mare and Other Animals," by Prof. Williams, of the New York State Veterinary College.

"Extraction of Teeth and Other Dental Operations," by Prof. Merillat, of the McKillip Veterinary College.

"Casting and Confining Animals," by Dr. Tait S. Butler.

---

The New York State Veterinary Medical Society will meet in New York City the week following the U. S. V. M. A. Convention

The Pennsylvania State Veterinary Medical Association will meet at Pittsburg on September 20th. A cordial invitation is extended to every veterinarian in Pennsylvania, Ohio, New York, Virginia, Maryland, and West Virginia, and they will be made very welcome.

The Keystone Veterinary Medical Association of Philadelphia and vicinity will meet at the usual place on Friday evening, September 16, 1898. This date has been selected so as not to conflict with those of the United States and State meeting dates.

A special meeting of the Alumni Association of the American Veterinary College will be held on September 22, 1898, at 3 P.M., at the College Building, for the purpose of taking the preparatory steps for a reunion of the alumni on the twenty-fifth anniversary of the College, in 1900. The President earnestly requests a full attendance that a proper celebration of this important event in the College's history may be consummated.

